# Zhujie Hewei Granules Ameliorated Reflux Esophagitis in Rats

**DOI:** 10.1155/2019/1392020

**Published:** 2019-12-24

**Authors:** Yue Qiu, Jia-liang Hu, Chun-cao Zhao, Ji-quan Zhang, Fei Wu, Bing-liang Ma, Yi Feng, Ke-feng Ruan

**Affiliations:** ^1^Engineering Research Center of Modern Preparation Technology of Traditional Chinese Medicine, Ministry of Education, Shanghai University of Traditional Chinese Medicine, Shanghai 201203, China; ^2^Department of Pharmacology, School of Pharmacy, Shanghai University of Traditional Chinese Medicine, Shanghai 201203, China; ^3^Shanghai Zhangjiang Engineering Research Center of Modern Preparation Technology of Traditional Chinese Medicine, Shanghai 201203, China

## Abstract

**Background:**

Gastroesophageal reflux disease (GERDs) is a common chronic digestive system disease, in which the symptoms of reflux esophagitis (RE) seriously affect the quality of life.

**Aims:**

We aimed to study the therapeutic effect of Zhujie Hewei granules (ZHG) on reflux esophagitis in model rats.

**Materials and Methods:**

A rat model of RE was established with the steps of half pylorus ligation, cardiotomy, and hydrochloric acid perfusion. The rats in treatment groups were orally administered with 1.30, 2.60, or 5.20 g/kg ZHG once daily for 28 days. Histopathological changes of the esophagus were observed with hematoxylin-eosin staining. The content of total bilirubin and pH in gastric juice was determined. Esophageal mucosal injury was assessed by macroscopic observation scores, mucosal injury index scores, and esophageal inflammation scores. The levels of gastrin (GAS), motilin (MTL), and vasoactive intestinal peptide (VIP) in serum were evaluated by using ELISA kits.

**Results:**

After treatment with ZHG, the body weight of RE rats tended to increase drastically, the macroscopic observation scores of the esophagus mucous membrane decreased (*P* < 0.05), the mucosal injury index scores decreased (*P* < 0.05), the gastric pH values increased (*P* < 0.05), and the levels of serum MTL and VIP decreased (*P* < 0.05). In addition, the high dose of the ZHG-treated group showed lower serum GAS (*P* < 0.05), while the high and middle doses of the ZHG-treated groups showed lower esophageal inflammation scores (*P* < 0.05).

**Conclusions:**

ZHG was effective in treating RE in rats due using mechanisms including improving the pH value of gastric contents, decreasing the gastrointestinal hormones (including GAS, MTL, and VIP), and improving the inflammatory damage.

## 1. Introduction

Gastroesophageal reflux diseases (GERDs) are the symptoms or complications resulting from the reflux of gastric contents into the esophagus, oral cavity (including larynx), or lung [1]. GERDs are common clinical chronic diseases that up to 40% of the world's population suffer from at least once a month [2]. The prevalence of gastroesophageal reflux in Asia is lower than that in Europe and America, whereas there is an increasing trend in Asia in recent years [3]. The reflux of gastric acid, bile salts, and other noxious agents contained in the refluxed gastric juice is regarded as the major cause of reflux esophagitis (RE) [4].

Proton pump inhibitors, antireflux drugs, gastric motility drugs, and antidepressants are usually used in clinics to treat GERDs. Proton pump inhibitor is the preferred drug of choice [2, 5]. It is the most potent in inhibiting gastric acid secretion and relieving the pain in patients with GERDs, but 20 to 30 percent of patients reported no significant results [6]. Additionally, proton pump inhibitors cannot reduce the key pathogenic factors in GERDs and cannot cure GERDs completely. Moreover, the long-term use of proton pump inhibitors leads to serious complications [7, 8].

Some traditional Chinese medicines (TCMs) had been used to treat GERDs, which were effective in relieving symptoms, increasing cure rate, reducing recurrence, and showing advantages in less adverse reactions [9–11]. Zhujie Hewei prescription, which consists of four herbs including *Eriobotryae folium*, *Atractylodis macrocephalae rhizoma*, *Gardeniae fructus*, and *Platycodonis radix*, is a clinically used TCM formula designed by the Department of Gastroenterology of Shanghai Traditional Chinese Medicine Hospital. It is effective in improving the symptoms of GERDs and has been engaged in the clinical treatment of GERDs for nearly 20 years and more than 10,000 outpatients. In addition, it is safe and causes no obvious adverse reactions in patients [12–14]. However, the therapeutic mechanism remains to be elucidated.

Here, we aimed to study the therapeutic effect of Zhujie Hewei granules (ZHG) derived from Zhujie Hewei prescription, on RE in rats, focusing on revealing the underlying mechanisms. The study would be helpful in promoting the clinical applications and new drug research and development of ZHG.

## 2. Materials and Methods

### 2.1. Materials

Omeprazole enteric-coated capsules (batch number 20160625) were purchased from Changzhou Siyao Pharmaceuticals Co., Ltd (Changzhou, Jiangsu, China). 2-0 and 3-0 sutures were purchased from Jinhuan Medical Supplies Co., Ltd, Shanghai, China. Microplate reader was purchased from BioTek Synergy 2. Gastrin (GAS) linked immunosorbent assay kit was purchased from Wuhan Myhalic Biotechnology Co., Ltd (Wuhan, Hubei, China). Motilin (MTL) linked immunosorbent assay kit was purchased from Wuhan Myhalic Biotechnology Co., Ltd (Wuhan, Hubei, China). Vasoactive intestinal peptide enzyme (VIP) linked immunosorbent assay kit was purchased from Wuhan Myhalic Biotechnology Co., Ltd (Wuhan, Hubei, China). Total bilirubin detection kit (chemical oxidase standard) was purchased from the Nanjing Institute of Bioengineering (Nanjing, Jiangsu, China).

### 2.2. ZHG Formulation

ZHG with batch numbers 20151218, 20151221, and 20151224 were prepared by Shanghai University of Traditional Chinese Medicine. ZHG was stored at room temperature and dissolved in water when used. ZHG was packed with 12.5 g per bag that was equivalent to 4.84 g *Atractylodis macrocephalae rhizoma*, 3.63 g *Eriobotryae folium*, 3.63 g *Gardeniae fructus*, and 0.40 g *Platycodonis radix*. In brief, the herbal piece of the TCMs was extracted three times with 10 times volumes of boiled water (1 h for each extraction). The extraction was then filtered and concentrated to a relative density of 1.14∼1.16. Finally, with dextrin as the base material, the concentrated solution is sprayed into the fluidized bed for drying and granulation to obtain ZHG. ZHG are stored in the environment of normal temperature and darkness, and the validity period is 24 months. ZHG are dissolved in water when used.

**Figure 1:**
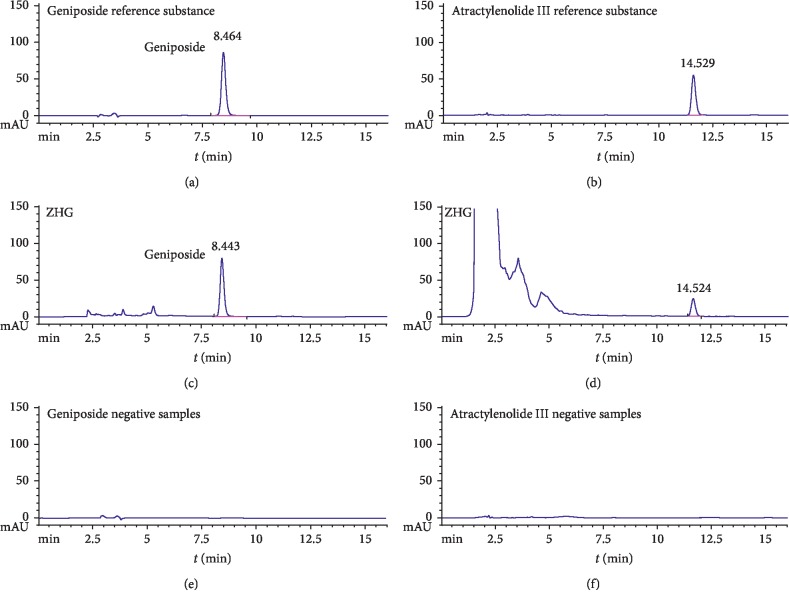
HPLC chromatography of geniposide and atractylode III.

The methods of evaluating the quality of ZHG were established using quantitative high-performance liquid cartography (HPLC). Geniposide in ZHG was determined using a HPLC method with the following conditions: Phenomenex C_18_ chromatographic column (4.6 × 250 mm, 5 *μ*m), mobile phase acetonitrile-water (85 : 15), flow rate 1.0 mL/min, detection wavelength 238 nm, and column temperature 30°C. In addition, atractylenolide III was measured using a HPLC method with the following conditions: Phenomenex C_18_ chromatographic column (4.6 × 250 mm, 5 *μ*m), mobile phase acetonitrile-water (50 : 50), flow rate 1.0 mL/min, detection wavelength 220 nm, and column temperature 30°C. The HPLC chromatogram of Zhujie Hewei granules is shown in Figure 1. The stability was investigated by HPLC every 0, 3, 6, 9, 12, 18, and 24 months. The average content of three ZHG batches (20151218, 20151221 and 20151224) of geniposide and atractylenolide III was 14.2 and 1.5 mg/g in ZHG, respectively. After 24 months of study of room temperature stability, the two indexes changed by −3.28%–14.38% and +5.60%–5.09%, respectively.

### 2.3. Animals

Sprague–Dawley rats (*n* = 98, half male and half female) with a body weight of 180–250 g were purchased from the experimental animal center of Lanzhou University. The rats were maintained in an experimental house under controlled temperature (22 ± 2°C) with a 12 h light-dark cycle and free access to water and a standard diet. All animal experimental protocols were approved by the Animal Care and Use Committee of Lanzhou University (Approval number 2013-0002). Prior to the experiments, all rats were given a period of at least 7 days for acclimatization.

### 2.4. Rat RE Model

The rat model of RE was prepared according to previous studies with modification [15–18]. In brief, the model was established with the steps of hemiplegia ligation, cardiotomy, and hydrochloric acid perfusion. Animals were deprived of food for 24 h prior to surgery. Anesthesia was administered using 0.4% sodium pentobarbital. The animal was tethered in a supine position, then the upper abdomen was entered through a 3 cm midline incision, and the pylorus of the rat was ligated by using 2-0 nonabsorbable suture. After that, the muscular layer was torn vertically upward about 0.70 cm at the esophagogastric junction and sutured again, and the abdomen was closed. Three days after the surgery, the rats were treated with 0.1 mol/L hydrochloride by gavage (1.0 mL/100 g), once a day for seven days. In the sham operation group, the stomach and duodenum were separated for 15 min without ligating the pylorus and duodenum, with water instead of hydrochloric acid by gavage.

### 2.5. Treatment

After the RE surgery or sham operation for 10 days, the rats were randomly divided into seven groups (*n* = 14 each group): normal control, sham, model (RE only), low-dose group of ZHG (RE + 1.3 g/kg ZHG), middle-dose group of ZHG (RE + 2.6 g/kg ZHG), high-dose group of ZHG (RE + 5.2 g/kg ZHG), and omeprazole (RE + 4.20 mg/kg omeprazole). The ZHG, omeprazole, or normal water (for the Normal control, Sham, and Model groups) was orally administered once a day by oral gavage for 28 days.

### 2.6. Determination of the Levels of the Gastrointestinal Hormones in Serum

The levels of gastrin (GAS), motilin (MTL), and vasoactive intestinal peptide (VIP) in serum were evaluated by using ELISA kits. The serum was separated by centrifugation at 3000×*g* (4°C) for 10 min. The serum was stored at −20°C before detection. The ELISA plate was read by using a microplate reader (BioTek Synergy 2). The concentrations were determined in accordance with the manufacturer's instructions and the standard curve. All the experiments were performed in triplicate.

### 2.7. Determination of the pH Value and Gastric Bilirubin Amount of the Gastric Contents

After the rats were euthanized, the cardia and pylorus were ligated and then, the whole stomach was taken out. After cutting along the side of the great curvature of the stomach, the gastric cavity was washed with 5 mL distilled water and the pH values of the supernatant of the gastric content were determined using a pH meter. After filtration with filter paper, the filtrate was centrifuged for 10 min, and the supernatant was taken to determine the amount of total bilirubin by detection kit. The supernatant was stored at −20°C before use in the bilirubin detection kit. Every sample was triplicate.

### 2.8. Histopathological Examination of Esophagus with H&E Staining

The lower esophagus of rats was dissected and cut open longitudinally. The tissue samples were fixed in 4% paraformaldehyde (Solarbio, Beijing, China) overnight at 4°C. The samples were then rinsed with PBS (Solarbio, Beijing, China) and embedded with paraffin (50–52°C, Solarbio, Beijing, China). Then the samples were serially sectioned at a thickness of 5 *μ*m for histologic analysis. The sections were stained with hematoxylin and eosin (Solarbio, Beijing, China). An optical microscope (Stemi DV4 or Axio Scope A1, Carl Zeiss, Oberkochen, Germany) was used to observe the histopathological changes. The index score of esophageal mucosa injury and the inflammation score of esophageal tissue were determined. The esophagus of the rat was cut longitudinally, and the damaged part was exposed, then the integral pathological grade of the esophagus ware calculated by the diagnostic criteria for RE, which was issued by the Chinese medical association digestive endoscopy society in February 1999 (Tables 1 and 2). Meanwhile, the degree of inflammation was also evaluated using the Harry S. Cooper inflammatory integral calculation method (Table 3).

### 2.9. Statistical Analysis

Data were presented as Mean ± SD. Statistically significant differences among groups were calculated by one-way ANOVA. Multiple recombination compartment comparisons were analyzed by using the LSD test. Dunnett's T3 test was used in the uneven variance. *P* < 0.05 was considered to be statistically significant.

## 3. Results

### 3.1. Effects of ZHG on Body Weight

**Figure 2:**
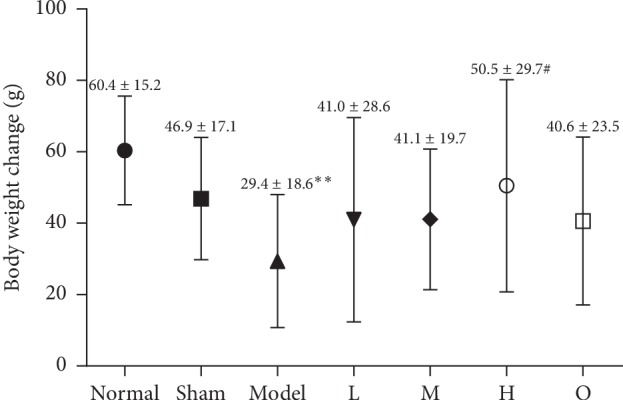
Effects of ZHG on body weight change.

As shown in Figure 2, the body weight of rats in the model group was significantly lower than those in sham operation groups (*F* = 2.603, *P*=0.041). Compared with the model group, ZHG increased body weight, as the treatment with a high dose of ZHG inhibited the body weight loss of the rats (*P*=0.16). In addition, compared with omeprazole, ZHG at middle and low doses had a better effect in inhibiting weight loss in rats (*P*=0.870; *P*=0.951).

### 3.2. Effect of ZHG on pH and Total Bilirubin

**Figure 3:**
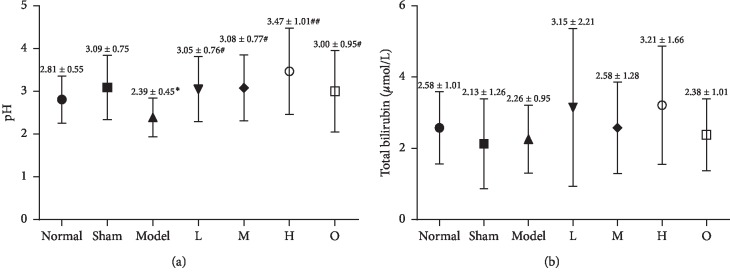
Effects of ZHG on ER-induced changes in pH and total bilirubin in the supernatant of gastric contents in rats.

As shown in Figure 3, compared to the sham group, the model group of reflux-induced esophagitis rats displayed a significant decrease in the gastric pH (*F* = 2.474, *P*=0.019). In contrast, the gastric pH in the low, middle, and high ZHG-treated groups was significantly increased (*P*=0.025; *P*=0.021; *P* < 0.00001). But there was no significant difference in the levels of total bilirubin between the normal group, the sham operation control group, the omeprazole group, and the high-, middle-, and low-dose groups of ZHG (all *P* > 0.05).

### 3.3. Effect of ZHG on the Histopathological Changes of Esophageal Mucosa

**Figure 4:**
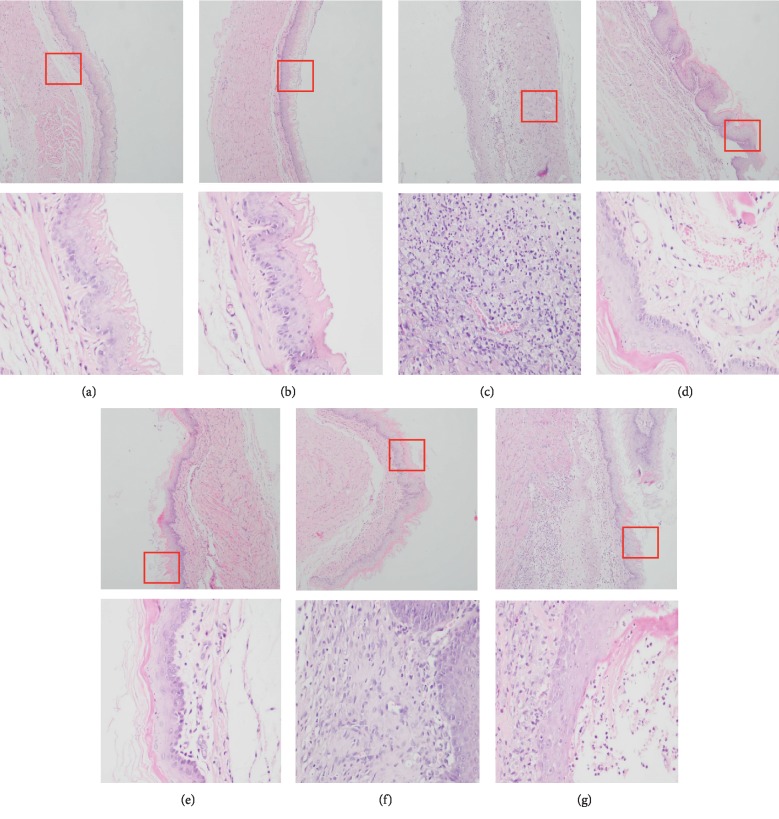
H&E showing pathological changes in rats. a–g, the surface of the rat's esophagus in the normal, sham operation, model, omeprazole, and high, middle, low dose of ZHG groups, respectively. (original magnification ×100 and 400).

**Figure 5:**
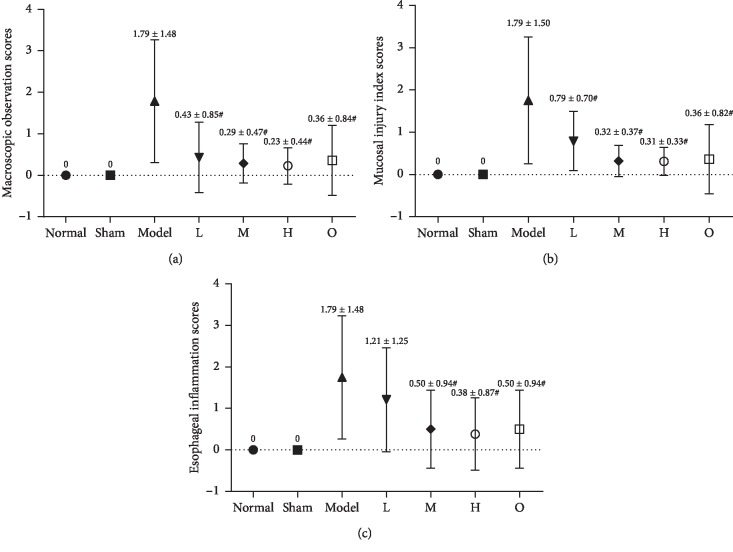
Effects of ZHG on macroscopic observation scores, mucosal injury index scores, and esophageal inflammation scores in rats.

As shown in Figure 4, the esophageal mucosa of rats in the normal and sham operation groups did not show any histological changes. In contrast, the epithelium and lamina propria of esophageal mucosa in the model group were necrotic and exfoliated, and a large amount of exuded cellulose, neutrophils, and necrotic tissue fragments were observed. The esophagus and mucosa of rats in the administration group were improved to varying degrees. As shown in Figure 5, the macroscopic observation scores of the esophagus mucosa of the rats in the ZHG groups and omeprazole groups were significantly lower than those in the model group (*F* = 9.136, *P* < 0.00001). There was no significant difference between ZHG groups and the omeprazole group in terms of the change of the macroscopic observation scores of the esophageal mucosa in the rats (*P*=0.805; *P*=0.805; *P*=0.668).

### 3.4. Scores of Esophageal Mucosal Injury Index in Different Groups

The esophageal mucosa of rats in the normal group and the sham operation control group was normal under a light microscope. As shown in Figure 5, compared with the model group, the scores of mucosal injury index in the low, middle, and high ZHG-treated groups were decreased, with statistical significance (*F* = 9.858, *P*=0.001; *P* < 0.00001; *P* < 0.00001). Moreover, there was no significant difference in esophageal mucosal injury index scores between ZHG groups and the omeprazole group (*P*=0.122; *P*=0.897; *P*=0.860).

### 3.5. Inflammation Scores of Esophagi in Different Groups

The results were shown in Figure 5. Compared with the model group, the inflammatory scores of esophageal tissue in the high and middle dose of ZHG-treated groups and omeprazole group decreased significantly (*F* = 6.656, *P*=0.001; *P* < 0.00001; *P* < 0.00001). The inflammation scores of the esophagus in the middle and high ZHG-treated groups were not different with the omeprazole treated group (*P*=1.000; *P*=0.753).

### 3.6. Effects of ZHG on GAS, MTL, and VIP

**Figure 6:**
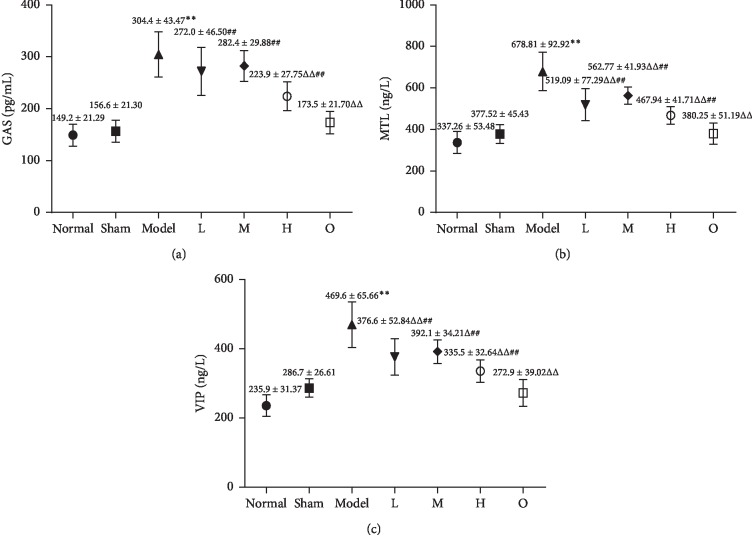
Effect of ZHG on GAS, MTL, and VIP in rats.

As shown in Figure 6, the serum levels of GAS, MTL, and VIP of rats in the model group were significantly higher than those in the sham operation group (*F* = 71.100, *P* < 0.00001; *F* = 56.145, *P* < 0.00001; *F* = 50.558, *P* < 0.00001). However, compared with the model group, the serum levels of these hormones in rats that received ZHG were significantly decreased (All *P* < 0.05 or *P* < 0.01).

## 4. Discussion

After oral ZHG, the body weight of rats increased, especially in the high dose group; the result shows that the ZHG is beneficial to the treatment of RE rats; the pH value of supernatant of gastric contents in rats increased, which indicated that ZHG had inhibitory effect on gastric acid and acid reflux. The secretion of total bilirubin has no obvious effect, which showed that ZHG will not affect the secretion of bile. The macroscopic observation scores, mucosal injury index scores, and esophageal inflammation scores were decreased, which presented that ZHG protected the mucosal injury and inflammatory damage; the levels of GAS, MTL, and VIP in serum of rats with reflux esophagitis were decreased; the result shows that the ZHG regulates the serum gastrointestinal hormone levels.

The determination of the pH value of the supernatant of gastric contents in rats showed that the pH value of the gastric contents of the model group was reduced, which was related to the model replicating method. The “semipyloric ligation” of the model could block the gastric emptying, increase the content of gastric contents, increase the fermentation, and produce acid [19–21]. Our results of pH test of rat gastric contents supernatant suggested that ZHG had an inhibitory effect on the release and/or reflux of gastric acid. However, the underlying mechanism is not clear yet. It may be related to the inhibition of gastric acid secretion and pepsin activity and promoting esophageal acid clearance.

In addition, the results showed that the levels of GAS, MTL, and VIP in the serum of rats with RE were increased but were decreased after treatment with ZHG. Gastrin (GAS) is the first gastrointestinal hormone that has been found to increase the lower esophageal sphincter pressure [22]. Motilin (MTL) is a hormone produced by the endocrine cells of the duodenal mucosa, which can promote the strong contraction of the stomach and accelerate the peristalsis of the intestine [23]. Vasoactive intestinal peptide (VIP), a kind of neurotransmitter, has dual functions of organisms, both gastrointestinal hormones and neuropeptides [24]. The change of GAS, MTL, and VIP levels are closely related to gastrointestinal diseases. By detecting the levels of GAS, MTL, and VIP in serum, we can judge the therapeutic effect of ZHG on RE rats. According to the results, ZHG can reduce the reflux degree of gastric contents in RE rats, reduce the inflammatory reaction and reduce the relaxation of the lower esophageal sphincter.

In the theoretical system of traditional Chinese medicine, GERDs are mainly manifested as acid reflux, stomach reflux, plum nucleus gas, chest arthralgia, and others [25]. The lesions of this disease are located in the stomach and the esophagus, which are closely related to the liver, the spleen, the lung, the kidney, and the gallbladder. Many factors such as diet and emotion lead to the dysfunction of the spleen and the stomach and then produce the symptoms of GERDs [26, 27]. ZHG applies traditional Chinese medicine theory of Yin Yang balance to syndrome differentiation and treatment. It is made up of four TCMs according to the law of compatibility of traditional Chinese medicine. The four TCMs complement each other and act on the human body together, which has achieved a good curative effect. Among them, *Eriobotryae folium* contains ursolic acid and oleanolic acid, which have obvious anti-inflammatory and gastroprotective effects and can alleviate the symptoms of RE [28, 29]; *Atractylodis macrocephalae rhizoma* is a popularly used TCM to treat gastrointestinal diseases. It contains atractylenolide III, which is the main gastric protective constituent [30]; *Gardeniae fructus* contains geniposide, which has the effects of anti-inflammation, regulating bile secretion and improving intestinal mucosal damage [31–33]; *Platycodonis radix* contains platycodon saponin D, which has a certain therapeutic effect on RE [34]. These components in the TCMs may work alone or synergistically to enhance the therapeutic effect. In brief, the therapeutic effects of ZHG may be attributed to the bioactive compounds discussed above but it still needs further research.

## 5. Conclusions

ZHG was effective in treating RE rats including improving the body weight, regulating the pH value in the stomach, protecting the mucosal injury and inflammatory damage, and regulating the GAS, MTL, and VIP levels of serum gastrointestinal hormone. Besides, as the TCM formula, Zhujie Hewei prescription has also been safely used clinically and achieved a curative effect without obvious adverse reactions in patients.

## Figures and Tables

**Table 1:** Esophageal histopathological score.

Gross manifestations of esophageal mucosa	Score
Normal (histologically changeable)	0
Point or stripe redness, erosion, no fusion phenomenon	1
Striped redness, erosion, fusion, nonholonomic	2
Extensive lesion, rubedo, total circumferential erosion, or ulcer	3

**Table 2:** Pathological grading of reflux esophagitis.

Grading		Pathology						Score
		Hyperplasia of squamous epithelium	Mucosal lamina propria papillae extension	Inflammatory cell infiltration in epithelial cell	Mucosal erosion	Exulceration	Barrett esophageal changes	
Normal		−	−	−	−	−	−	0
Mild		+	+	+	−	−	−	1
Medium		+	+	+	+	−	−	2
Severe	+	+	+	−	+	+/−	3

**Table 3:** Analysis of inflammatory cells in the esophagus.

Intensity and extent of inflammation	Score
There is almost no infiltration of inflammatory cells in the lamina propria	0
There are a small number of inflammatory cells in the lamina propria and no infiltration of inflammatory cells in the epithelial layer	1
There are more inflammatory cells in the lamina propria and no infiltration of inflammatory cells in the epithelial layer	2
There are a lot of inflammatory cells in the lamina propria and a small number of inflammatory cells infiltrate into the epithelial layer	3
Lymphoid nodules were formed in the lamina propria and a large number of inflammatory cells infiltrated in the epithelial layer	4

## Data Availability

The data used to support the findings of this study are included within the article.
